# Genetic and environmental factors of schizophrenia and autism spectrum disorder: insights from twin studies

**DOI:** 10.1007/s00702-020-02188-w

**Published:** 2020-04-13

**Authors:** Akira Imamura, Yoshiro Morimoto, Shinji Ono, Naohiro Kurotaki, Shinji Kanegae, Naoki Yamamoto, Hirohisa Kinoshita, Takahiro Tsujita, Yuji Okazaki, Hiroki Ozawa

**Affiliations:** 1 Child and Adolescent Psychiatry Community Partnership Unit, Nagasaki University Hospital, Nagasaki, Japan; 2 Unit of Translation Medicine, Department of Neuropsychiatry, Nagasaki University Graduate School of Biomedical Sciences, Nagasaki, Japan; 3 Department of Human Genetics, Atomic Bomb Disease Institute, Nagasaki University Graduate School of Biomedical Sciences, Nagasaki, Japan; 4 Department of Clinical Psychiatry, Graduate School of Medicine, Kagawa University, Kita-gun, Japan; 5 Goseikai Hironaka Hospital, Nagasaki, Japan; 6 Koseikai Michinoo Hospital, Nagasaki, Japan; 7 Tokyo Metropolitan Matsuzawa Hospital, Tokyo, Japan

**Keywords:** Schizophrenia, Autism spectrum disorder, Twin study, Psychiatric disorders, Epigenetics

## Abstract

Twin studies of psychiatric disorders such as schizophrenia and autism spectrum disorder have employed epidemiological approaches that determine heritability by comparing the concordance rate between monozygotic twins (MZs) and dizygotic twins. The basis for these studies is that MZs share 100% of their genetic information. Recently, biological studies based on molecular methods are now being increasingly applied to examine the differences between MZs discordance for psychiatric disorders to unravel their possible causes. Although recent advances in next-generation sequencing have increased the accuracy of this line of research, there has been greater emphasis placed on epigenetic changes versus DNA sequence changes as the probable cause of discordant psychiatric disorders in MZs. Since the epigenetic status differs in each tissue type, in addition to the DNA from the peripheral blood, studies using DNA from nerve cells induced from postmortem brains or induced pluripotent stem cells are being carried out. Although it was originally thought that epigenetic changes occurred as a result of environmental factors, and thus were not transmittable, it is now known that such changes might possibly be transmitted between generations. Therefore, the potential possible effects of intestinal flora inside the body are currently being investigated as a cause of discordance in MZs. As a result, twin studies of psychiatric disorders are greatly contributing to the elucidation of genetic and environmental factors in the etiology of psychiatric conditions.

## Introduction

Twins have long been a source of mystery. In Greco-Roman mythology, the twin-pair of one human and one immortal (Castor and Pollux; Gemini) served as an important example of “discordant” twins. At the end of the nineteenth century, Francis Golton was the first to consider twins as a useful scientific model, questioning whether human traits originated from genetic or environmental causes. This line of inquiry led to the “nature versus nurture” debate that is still argued to this day (Torrey et al. 1994a).

In epidemiological twin studies, comparisons of the concordance rate between monozygotic twins (MZs) and dizygotic twins (DZs) are very important. Since MZs are considered to share 100% of their genetic information, their concordance rate is higher compared to DZs who share about 50% of the genetic information. Thus, genetic factors are thought to play a major role as compared to environmental factors (Rutter 2006). In psychiatry, there is a much higher concordance rate for schizophrenia (SCZ) and autism spectrum disorder (ASD) in MZs versus DZs (Hilker et al. 2018; Sandin et al. 2017). As a result, researchers are beginning to think that genetic factors play a major role in the onset of these conditions.

The similarity of the genetic information in MZs provides a useful means for determining the disorder-causing role of environmental factors. In recent years, studies on the differences between genomic and epigenomic characteristics of MZs have led to the development of new approaches for elucidating the etiology of psychiatric disorders (Liang et al. 2019; Morimoto et al. 2017). The current paper focuses on epidemiological and molecular genetic research studies in MZs with the purpose of unravelling useful insights on factors that can lead to SCZ and ASD.

## Epidemiological twin studies

### Studies on heritability

#### Heritability of SCZ

In 1899, Emil Kraepelin classified psychosis into ‘dementia praecox’ [Schizophrenia in DSM-5 (American Psychiatric Association 2013)] and ‘manisch-depressiven Irreseins’ (Bipolar Disorder in DSM-5). This has had a significant impact on subsequent diagnostic classifications. At that time, the idea of urbanization and mental stress as factors that could cause psychosis in young people was widely accepted. However, Kraepelin argued for the involvement of a biological component (Kendler and Engstrom 2018). Eugen Bleuler agreed with Kraepelin’s theory but considered hallucinations and delusions to be secondary with conditions resulting from some disruption in the cognitive processing regarded as psychopathologies (Maatz and Hoff 2014).

In the 1940s, the idea that SCZ was due to mother–child relationships was widely accepted with Fromm-Reichman’s term, “schizophrenogenic mother”, emphasizing the role that the environment played in the onset of SCZ (Fromm-Reichman 1948). However, epidemiological studies such as adoption studies, twin studies, and high-risk studies support the idea that genetic factors play a significant role with regard to the cause of SCZ (Henriksen et al. 2017).

In SCZ twin studies, MZs have been reported to exhibit a much higher concordance rate compared to DZs. Researchers have used two statistical methods to examine the twin concordance rate: the probandwise method and the pairwise method. The pairwise method is able to detect twin pairs with one or two affected twins while the probandwise method evaluates each twin as a distinct target. When using the latter method, if both twins are detected with the condition, it is possible that the same twin may be counted twice. Hence, this approach produces a higher concordance rate as compared to the pairwise method (Torrey et al. 1994b). Currently, the probandwise method is more commonly used by researchers. Using the probandwise method, Farmer et al. (1987) reported SCZ concordance rates in the DSM, Third Edition (DSM-III) were 47.6% and 9.5% for MZs and DZs, respectively. Onstad et al. (1991) examined the MZ and DZ concordance rate and reported them to be 48.0% and 4.0%, respectively, for SCZ in the DSM-III Revised Edition (DSM-III-R).

In a large-scale survey of twins in Nagasaki Prefecture that was conducted to investigate the DSM-III-R SCZ concordance rate in Japan, Okazaki (1995) reported a concordance rate of 11/22 (50.0%) when using the probandwise method, 7/18 (38.9%) when using the pairwise method for MZs, and 1/7 (14.0%) for both methods for DZs. During the same period, Torrey (1992) evaluated the results from eight studies and reported that the concordance rate among MZs with SCZ was 163/405 (40.2%) and 97/341 (28.4%) when using the probandwise and pairwise methods, respectively. For DZs, results were 62/427 (14.5%) and 36/587 (6.1%), respectively, when using the probandwise and pairwise methods.

A recent study by Hilker et al. (2018) used data from the Danish Twin Registry and Danish Psychiatric Registry to examine the concordance rate of SCZ and its spectrum (F2 code ICD-10) in more than 30,000 twin pairs. When using the probandwise method, they found the SCZ concordance rate for MZs and DZs was 33% and 7%, respectively. The SCZ heritability rate was 79% while the heritability rate of the SCZ spectrum disorders was 73%.

Since the SCZ twin concordance rate was much higher in MZs compared to DZs, this clearly shows that there is involvement of a genetic component in the disorder onset.

#### Heritability of ASD

Kanner (1943) examined eleven autism cases and suggested that they could potentially be congenital disorders. He also recorded the characteristic personalities of the parents, stating that they were intelligent, had a moderately high social status, while they were unsociable, emotionally fervent, compulsive, unwarm, and unemotional towards their children. In contrast, Asperger (1938, 1944) hypothesized there was a predisposition for autism to be passed on from parent to child, a theory implying multiple-factor inheritance. Thus, over a long period of time, Kanner’s remark has led to the wrong assumption and the commonly accepted view that autism was the result of the mother–child relationship. Moreover, a large number of researchers believed that being raised in an environment similar to the cold parenting style described by Kanner predisposed children to autism. Bruno Bettelheim described this term as “refrigerator mother”, with this notion then strongly adopted by the psychoanalysis field (Mandy and Lai 2016).

Starting in the 1970s, Rutter et al. argued that autism was, in fact, a congenital cognitive disorder caused by strong genetic factors, which marked the eventual end to theories suggesting psychological or environmental factors were primarily responsible for causing autism (Rutter and Bartak 1971). In addition, twin studies have also shown that autism is associated with biological factors, especially those of genetic origin. Folstein and Rutter (1997) analyzed 21 twin pairs and reported that the MZs concordance rate for autism was 36% (82%, if broader phenotypes were included), while the DZs rate was 0% (10%, if broader phenotypes were included) when using the pairwise method. The heritability rate was calculated to be 91–93% in this group. Subsequent works by Rutter et al. demonstrated that autism was a disorder with a particularly high heritability rate. Later, the concept of “autism spectrum disorder (ASD)” was developed to broaden the diagnosis and which included the “Kanner type” and “Asperger type” (Wing 1996).

In 2011, Hallmayer et al. calculated the heritability rate for 192 pairs of twins and reported a heritability rate of 37% (95% CI 8–84%) for autism and 38% (95% CI 14–67%) for ASD. The heritability of autism and ASD with shared environmental factors was 55% (95% CI 9–814%) and 58% (95% CI 30–80%), respectively. Since the heritability rate of autism was shown to be much lower than expected, this led the researchers to reconsider the notion of the heritability of autism.

In 2017, Sandin et al. conducted a study of 37,570 twin pairs, 2,642,064 full sibling pairs, 432,281 maternal half-sibling pairs, and 445,531 paternal half-sibling pairs. They found that the heritability rate for ASD was 83% (95% CI 79–87%) and the ASD heritability rate from twin studies alone was 87% (95% CI 68–96%). These results demonstrated the greater influence of genetic factors compared to environmental factors.

Currently, a large percentage of researchers consider that ASD is one of the highest heritability rate common disorders.

#### Challenges in heritability research

**Fig. 1:**
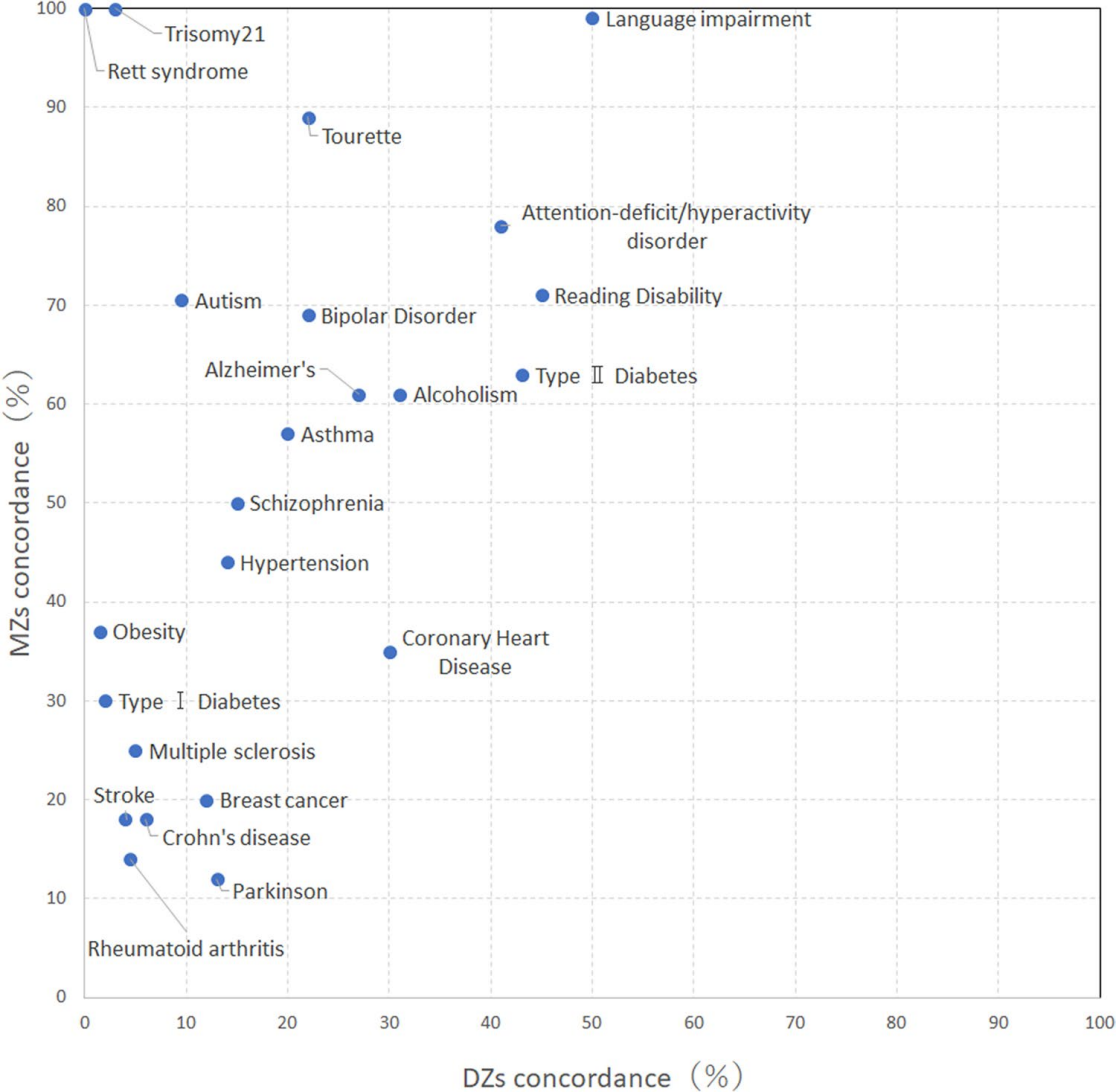
Concordance rate of MZs and DZs for various disorders. Originally cited and published in the study of Schumacher and Petronis (2006). We have added data on ADHD (Pingault et al. 2015) to the original graph that is pictured here

Several heritability studies have been carried out for different disorders. Figure 1 shows the concordance rate for MZs and DZs in these various disorders. Results suggested that the concordance rate for MZs in ASD and SCZ is much higher than the concordance rate for DZs.

Generally, the heritability rate is calculated from the correlation coefficient “rMZ” for MZs and “rDZ” for DZs. It is represented by the following equation:

$${\text{h2 }} = { 2}\left( {{\text{rMZ}} - {\text{rDZ}}} \right),$$

where h2 = heritability rate, rMZ = MZ concordance rate and rDZ = DZ concordance rate.

**Fig. 2:**
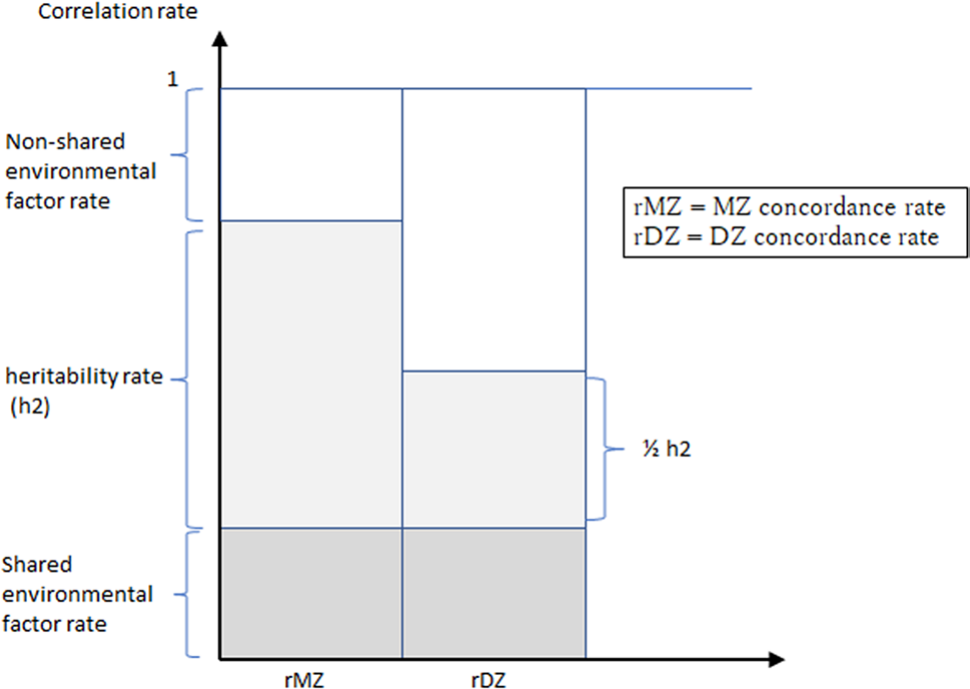
Heritability and shared environmental factor. This figure cites and was created based on the study by Hrubec and Neel (1981)

Figure 2 shows the relationship between the heritability and shared environmental factor.

However, the problem when using this method is that the diagnostic concordance rate varies depending on how the affected and non-affected twins are determined. For instance, the designation for autism advocated by Kanner was significantly different from the current ASD diagnostic criteria. Moreover, it has been reported that when using the broader autistic phenotype principle, this makes the concordance rate more variable (Le Couteur et al. 1996).

Furthermore, not only the heritability rate but both the “shared environmental factors” (rMZ-h2) and “non-shared environmental factors” (1-rMZ) need to be considered (Hallmayer et al. 2011). Although the intrauterine environment has previously been considered to be a shared environment, it has now been reported that studies of MZs cannot confirm that the intrauterine state is the same. An example of this is the twin transfusion syndrome (Djaafri et al. 2017). Moreover, if the epigenetic mutations or somatic mosaic mutations are acquired by one twin, the twins will then become “discordant.” Such factors are considered to be a part of the non-shared environment and disregarding these will only create different forms of misunderstanding. Recently, epigenetic changes experienced by parents due to environmental factors have been reported to be “transgenerational epigenetic inheritance” such as germ line-inherited H3K27me3 (Zenk et al. 2017), which makes it difficult to separate genetic and environmental factors (Nagy and Turecki 2015).

### Genetic overlap

Genetic overlaps have been recently observed between various mental disorders. In the 2010s, researchers examined the genetic background and overlap for SCZ, ASD, attention-deficit/ hyperactivity disorder (ADHD), bipolar disorder, depression, and other mental disorders (Cross-Disorder Group of the Psychiatric Genomics Consortium 2013; Zhao and Nyholt 2017; Gandal et al. 2018).

Epidemiological twin studies have also reported finding such genetic overlaps. Taylor et al. (2015) studied the genetic overlap between the ASD trait (autism spectrum condition (ASC)) and the SCZ trait (psychotic experiences). In their study of approximately 5000 twin pairs in the UK, ASC was weakly correlated with psychotic experiences (paranoia and hallucinations) and modestly correlated with cognitive disorganization in adolescence.

Another twin study attempted to investigate the genetic overlap between SCZ and bipolar disorder (Johansson et al. 2019) while another twin study showed a strong correlation between ASD symptoms (especially restrictive repetitive behaviors) and ADHD symptoms (inattention and hyperactivity-impulsivity) (Polderman et al. 2014). These studies suggest the existence of a genetic overlap in MZs with psychiatric disorders.

## MZ studies of molecular genetics

### Genomic differences

Originally, discordant MZs have been used to investigate the effect of environmental factors from the point of view that if MZs with the same genetic information exhibited different phenotypes, it might be due to various environmental factors. Since the 1980s, chromosomal abnormalities that resulted in different phenotypes have only been reported to occur in one individual from an MZ pair [Ring chromosome 18 syndrome (Hata et al. 1982)]. In other similar cases, MZs with phenotype differences due to genomic discrepancies, such as the genomic printing mechanism collapse [Beckwith–Wiedemann syndrome (Weksberg et al. 2002) and others], repeat sequences expansion [Fragile × syndrome (Kruyer et al. 1994) and others] have also been observed.

In 1990s, the earliest MZs genetic studies applied the restriction enzyme method in which DNA fragments were treated with restriction enzymes and then run on an electrophoresis gel for analysis. The use of DNA microarray technology allowed scholars to determine differences in the genomes of the discordant MZs. Kakiuchi et al. (2003) of RIKEN Brain Science Institute, Japan collaborated with our lab to conduct DNA microarray (Affymetrix Hu95A Chip, including over 12,600 probes) on two pairs of discordant bipolar cases. They found that a single nucleotide polymorphism (SNP) on the XBP1 gene. XBP1 is involved in the endoplasmic reticulum stress response, and genopolymorphic XBP1 has been shown to increase the risk of bipolar disorder. Kakiuchi et al. (2008) also conducted microarray experiments in two cases of MZs with SCZ (Affymetrix HU133A, 22,000 probes) and found SNP mutations in the adrenomedullin and sepx1 genes, which might be biomarkers of SCZ.

Since the late 2000s, studies on the copy number variation (CNV) in mental disorders have been ongoing, particularly for SCZ and ASD. Therefore, twin studies have been used to investigate CNV. In 2010, Ono et al. conducted microarray experiments on three MZ SCZ polymorphisms (AffymeTrix Genome-Wide Human SNP Array 6.0; 906,600 or more SNP probes, including 946,000 copy count probes). They examined SNPs and CNVs between twins. The SNP analysis showed 36 loci with the potential loss of heterozygosity in affected twins while the copy number analysis revealed 120 loci with potential CNV differences. Ono et al. (2010) further conducted direct DNA sequencing and quantitative PCR and found no genomic alternations between twins. They concluded that the phenotype mismatch among MZs most likely involved epigenetic changes as a result of environmental events.

In 2017, Morimoto et al. conducted whole-exome sequencing on three MZ cases involving familial SCZ, one MZ case with ASD, and one MZ case with a gender identity disorder. A next-generation sequencer was used to produce deep sequencing and revealed three affected alleles between the twins with the gender identity disorder. It was hypothesized that these alleles were the result of somatic cell mosaicism that occurred during development, which indicated that mosaicism is an important mechanism in the generation of MZ differences.

Although the development of deep sequencing technology has enabled such new discoveries, the majority of differences between MZs are believed to be due to epigenetic mutations, as it is rare for somatic cell mutations to cause phenotype differences.

### Epigenomic differences

The term epigenetics refers to postnatal modifications of gene expression without changes of DNA sequences. DNA methylation, chemical modification of histone proteins, non-coding RNA, and other mechanisms are involved in epigenetic regulation. Epigenetic factors are believed to play a vital role in human twin variation, in addition to causing a variety of diseases such as cancer, when abnormally regulated (Tabatabaiefar et al. 2019).

Another concept related to epigenetics is the concept of the Developmental Origins of Health and Disease (DOHaD). DOHaD considers various environmental factors from the fetal period to the developmental period that subsequently affect health in adulthood and other post-developmental factors that influence the onset of a disease by an epigenetic mechanism. Mental disorders are also believed to be caused by the same mechanism (O'Donnell and Meaney 2017).

In one of the earliest epigenetic studies of psychiatric disorders, Tsujita et al. (1998) used the restriction landmark genome scanning method to investigate the genomes of MZs, one with SCZ and the other without any health issues. The authors identified two spots out of nearly 2000 spots that were potential indicators of biological differences between the twin-pairs. Since they used the methylation-sensitive Not1 enzyme, they speculated some postzygotic events could lead to epigenetic DNA modification in one twin that may also account for the observed phenotypic variation.

Later, many researchers started using “bisulfite” to differentiate cytosine and methylated cytosine in studies that examine DNA methylation (Kinoshita et al. 2013; Liang et al. 2019). With bisulfite, cytosine is deaminated and converted to uracil, while methylated cytosine is left in its original state. In many studies, the use of bisulfite made it possible to compare the state of DNA methylation between twins. Recent research has focused on the use of microarrays in genome-wide studies, which are referred to as an epigenome-wide association study.

In a study on epigenetic changes in SCZ discordant MZs, Castellani et al. (2015a, b) used a NimbleGen Methylation Promoter Microarray to examine differences in the DNA methylation between epigenomes of twins. Their results found three sets of gene clusters in the DNA methylation and two common networks that were potentially responsible for the onset of SCZ. These types of experiments are considered to be useful when studying SCZ etiology.

Table 1 shows the history of genetic and epigenetic discordant MZ studies for SCZ and ASD. The results of the technological progress in these research fields that are in many of the studies suggest that discordant MZs sometimes exhibit discordant genetic or epigenetic features. However, these findings were not definitively confirmed.

**Table 1:** Genetic or epigenetic twin studies for schizophrenia (SCZ) and autism spectrum disorder (ASD)

References	Diagnosis	Genetics/epigenetics	Study aims	Number of discordant MZs	Methods (platforms)	Results
Wahlström et al. (1989)	ASD	Genetics	Detection of chromosomal abnormalities	1	Chromosome analysis	(−)
Polymeropoulos et al. (1993)	SCZ	Genetics	Search for the occurrence of a genetic event, such as a postzygotic mitotic crossover	5	Genetic analyses using 94 microsatellite repeat polymorphic markers	(−)
Brando et al. (1996)	SCZ	Genetics	Detection of the expansions of dentatorubral-pallidoluysian atrophy triplet repeat in the CTG-B37 region	26	Sequencing of dentatorubral-pallidoluysian atrophy CAG repeats	(−)
Tsujita et al. (1998)	SCZ	Epigenetics	Search for the occurrence of epigenetic DNA modifications (methylation-sensitive enzyme *Not*I restriction fragment)	1	Restriction landmark genome scanning analysis	(+ −)
Vincent et al. (1998)	SCZ	Genetics	Search for expanded CAG/CTG repeats	12	Repeat expansion detection and locus-specific PCR	(−)
Deb-Rinker et al. (1999)	SCZ	Genetics	Search for differences in the sequences of retroviral origin	3	Representational difference analysis	(+ −)
McDonald et al. (2003)	SCZ	Genetics/ epigenetics	Detection of genetic (DNA sequence) or epigenetic (HpaII site methylation) differences between discordant MZs	1	Representational difference analysis	(−)
Nguyen et al. (2003)	SCZ	Genetics	Detection of differences in the targeted genomic differential display peak similarity around (CAG)(n) repeating sequences	4	Targeted genomic differential display	(−)
Murphy et al. (2005)	SCZ	Epigenetics	Search for DNA methylation of the COMT promoter regions	3	Analysis of cytosine DNA methylation profile (sodium bisulfite conversion of genomic DNA)	(−)
Cannon et al. (2005)	SCZ	Genetics	Detection of differences in the sequence variations in the DISC1 and TRAX genes	20	DNA analysis of 3 single-nucleotide polymorphic markers and a rare haplotype incorporating 4 markers (DISC1 and TRAX genes)	(+ −)
Hu et al. (2006)	ASD	Gene expression	Detection of differential gene expression patterns in the DNA microarrays	3	Analysis of differential gene expression (TIGR 40 K Human Set)	(+ −)
Kakiuchi et al. (2008)	SCZ	mRNA expression pattern	Examination of the mRNA expression pattern	2	Analysis of mRNA expression (Affymetrix HU133A chip)	(+ −)
Murphy et al. (2008)	SCZ	Epigenetics	Detection of differences in the DNA methylation and mRNA expression of SYN III	1	Sequencing of sodium bisulfite amplified PCR (Applied Biosystems 377 ABI Automatic Sequencer)	(−)
Ono et al. (2010)	SCZ	Genetics	Detection of differences in the copy number variations and gene mutations	3	Genome-wide copy number variation analysis (Affymetrix Genome-Wide Human SNP Array 6.0)	(−)
Sarachana et al. (2010)	ASD	Epigenetics	Examination of global miRNA expression profiling	3	Analysis of miRNA expression (Custom-printed miRNA microarrays)	(+ −)
Nguyen et al. (2010)	ASD	Epigenetics	Examination of the global methylation profiling	3	Global methylation analysis (CpG island microarray analysis)	(+ −)
Maiti et al. (2011)	SCZ	Genetics	Detection of differences in the de novo copy number variations and single nucleotide polymorphism (SNPs) between twins	2	Genome-wide copy number variations and SNPs analysis (Affymetrix 6.0 human SNP array)	(+ −)
Dempster et al. (2011)	SCZ, BD	Epigenetics	Detection of differences in the DNA methylation	SCZ 11, BD 11	Genome-wide analysis of DNA methylation (Illumina Infinium HumanMethylation27 BeadChip)	(+ −)
Bönsch et al. (2012)	SCZ	Epigenetics	Detection of differences in the global DNA methylation (HpaII/MspI site) and promoter (Reelin and SOX10 genes) specific methylation	8	Analysis of methylation of genomic DNA and promoter methylation of Reelin and SOX10 genes	(+ −)
Rio et al. (2013)	ASD	Genetics	Detection of differences in the chromosomal anomalies	1	Molecular karyotyping (comparative genomic hybridization (CGH) arrays: CytoChips microarray and the Agilent 244 K oligonucleotide microarrays)	(+ −)
Kinoshita et al. (2013)	SCZ	Epigenetics	Detection of differences in the DNA methylation	3	Genome-wide DNA methylation profiling of peripheral leukocytes (Infinium HumanMethylation450 BeadChip)	(+ −)
Bloom et al. (2013)	SCZ, BD	Genetics	Search for differences in the copy number variation	SCZ 3, BD 2	Genome-wide copy number variation analysis (Roche Nimblegen 2.1 M probe CGH array)	(−)
Miyake et al. (2013)	Rett syndrome (ASD)	Genetics/ epigenetics	Comparison of genomic and epigenomic expression	1	Whole genome sequencing (Illumina HiSeq2000 sequencers) and genome-wide DNA methylation analysis (Infinium HumanMethylation450 BeadChip)	(+ −) (epigenetics)
Laplana et al. (2014)	ASD	Genetics	Search for differences in the copy number variation	1	Genome-wide copy number variation analysis (Agilent 400 K CGH array)	(−)
Wong et al. (2014)	ASD	Epigenetics/ genetics	Investigation of ASD-associated epigenetic variation and copy number variation	ASD 6, ASD traits 28	Genome-wide microarray analysis using bisulphite pyrosequencing and genome-wide copy number variation analysis (Illumina HumanOmniExpress BeadChip)	(+ −)
Castellani et al. (2014)	SCZ	Genetics	Search for differences in the copy number variation	6	Genome-wide copy number variation analysis (Affymetrix® Human SNP 6.0 arrays)	(+ −)
Castellani et al. (2015a, b)	SCZ	Genetics/ epigenetics	Detection of differences in the genome sequencing and DNA methylation	2	Whole genome sequencing and genome-wide methylation analysis (NimbleGen Human DNA Methylation 3 × 720 k CpG Island Plus RefSeq Promoter Microarray)	(+ −)
Fisher et al. (2015)	Childhood psychotic symptoms	Epigenetics	Detection of differentially methylated positions associated with psychotic symptoms	18 pairs at age 5 and 24 pairs at age 10	Genome-wide methylation analysis (Infinium HumanMethylation450) BeadChip array	(+ −)
Lyu et al. (2016)	SCZ	Genetics	Exploration of the novel pathogenic somatic single nucleotide variations and somatic insertions and deletions (indels)	1	Whole exome sequencing (Illumina HiSeq2500 sequencing platform)	(−)
Morimoto et al. (2017)	SCZ, ASD, GID	Genetics	Identification of discordant variants including somatic mosaic mutations	SCZ3, ASD1, GID1	Deep sequencing analysis (Illumina HiSeq2500)	(+ −) in GID
Li et al. (2017)	SCZ	Genetics (mitochondrial DNA)	Detection of mitochondrial DNA (mtDNA) heteroplasmy	8	Whole mtDNA genome sequencing	(−)
Tang et al. (2017)	SCZ	Genetics	Detection of association between de novo mutations and etiology of SCZ	8	Whole genome sequencing analysis	(+ −)
Castellani et al. (2017)	SCZ	Genetics	Identification of genome-wide post-zygotic mutations	2	Genome sequencing platform by Complete Genomics, Inc	(+ −)
Nishioka et al. (2018)	SCZ	Genetics	Exploration of somatic mutations	4	Whole exome sequencing (Illumina HiSeq 2000)	(+ −)
Huang et al. (2019)	ASD	Genetics	Comprehensive scan of genomic differences	3	Whole exome sequencing (Illumina HiSeq 2000)	(+ −)
Liang et al. (2019)	ASD	Epigenetics	Detection of differences in the DNA methylation	5	Genome-wide DNA methylation analysis (Infinium HumanMethylation450 BeadChip (450 K) Array)	(+ −)

If the cited authors completely achieved the study aims and the results of the study have been supported in the literature, the Results column shows (+). If the cited authors achieved a part of the study aims, the Results column shows (+ −). If the cited authors failed to achieve any of the study aims, the Results column shows (−)

*MZs* monozygotic twins, *SCZ* schizophrenia, *ASD* autism spectrum disorder, *BD* bipolar disorder, *GID* gender identity disorder

Future research into the epigenetic differences between twins is expected to heavily focus on histone chemical modifications and microRNA activity (Sarachana et al. 2010).

### Recent issues

In twin studies of SCZ and ASD, attempts have been made to identify the cause of onset from differences in neuroimaging research of MZs. MRI studies showed that twins with SCZ had a smaller hippocampus, thalamus, prefrontal cortex and various other areas as compared to the healthy co-twins (Besteher et al. 2020). Other researchers examining twins reported smaller white matter volumes were related to SCZ (Picchioni et al. 2017; Hulshoff Pol et al. 2012). Recently, diffusion tensor imaging studies have revealed impairment of the white matter that was related to the deficits of the oligodendrocyte and cognitive dysfunction in SCZ patients (Camchong et al. 2009). Furthermore, other molecular studies have shown that oligodendrocyte-related genes were differentially expressed in patients with SCZ (Åberg et al. 2006; Haroutunian et al. 2007; Kerns et al. 2010; Raabe et al. 2019). As described above in the recent twin research, the number of studies integrating not only neuroimaging but also brain functions and molecular genetic findings have been increasing.

With regard to the results of environmental factors in twin studies, various perinatal factors, such as low birth weight, hypoxia, and jaundice, are thought to affect the development of SCZ and ASD (Torrey et al. 1994c; Froehlich-Santino et al. 2014). Perinatal hypoxia is especially considered to be one of the most important factors, as it can cause brain damage related to myelin dysfunction that is the result of oligodendrocyte impairment or inflammation involving microglia (van Tilborg et al. 2018; Picchioni et al. 2017). Other recent studies using genetic or epigenetic methods have also been conducted to verify that prenatal hypoxia plays a role in the development of SCZ (Schmidt-Kastner et al. 2012; Palma-Gudiel et al. 2019).

In recent years, studies on intestinal flora have been ongoing in connection with epigenetic investigations. Many of these studies have focused on the relationship between ASD and the intestinal flora (Mangiola et al. 2016; Ding et al. 2017). In MZs, the intestines are completely sterile before birth, but are soon colonized by a large number of bacteria following delivery. The difference in the intestinal environments between MZs depends on several factors, including breast-fed versus formula-fed, vaginal versus C-section birth, maternal stress, developmental environment, and drugs taken by the mother. Although intestinal floras are clearly individual factors, they can be classified as environmental factors. These types of factors have not been previously taken into consideration. With future work on different intestinal environments between MZs, there is room for the development of a hypothesis built on epigenetic factors related to the immune system that is influenced by intestinal floras with regard to the etiology of ASD and SCZ.

One of the most difficult problems in epigenetic research for mental disorders is that the epigenome differs significantly depending on the tissue type. Since these types of experiments can only examine the DNA methylation extracted from peripheral blood cells, researchers have been previously criticized for neglecting the actual brain tissue. In response to this, DNA methylation has been investigated in postmortem brain tissues. However, various issues are encountered when studying postmortem brains including, the difficulty in obtaining a discordant twin sample, various epigenetic states being found in each part of the brain, brain tissue damage, and epigenetic changes due to lack of oxygen at the time of death.

For the purpose of solving such issues, studies using induced pluripotent stem (iPS) cells have been conducted. Neuronal iPS cells are established from skin cells or peripheral blood-derived cells and then induced to form neurons or glial cells. In one SCZ twin study, Nakazawa et al. (2017) discovered a MZ who had a mismatched clozapine response and was then able to establish iPS cell lines for both twins. Upon comparing the transcriptome profiles following treatment with clozapine, results showed the expression patterns differed between the iPS cell-derived differentiated neurons of the twins.

Recently, human pluripotent stem cells such as iPS cells have been used in three-dimensional (3D) culture techniques to create “organoids” that can reproduce the characteristics of certain small areas of human organs. Organoids can also be used to observe cell interactions in organs and within an extracellular environment. Although not a twin study, Mariani et al. (2015) developed iPS cells derived from patients with idiopathic ASD. Telencephalic organoids derived from iPS cells showed overexpression of the transcription factor FOXG1 and the overproduction of GABAergic neurons. Thus, by examining gene expressions of organoids, this can assist in the examination of causes of psychiatric disorders.

For mental disorders such as SCZ and ASD, it is believed that there is a genetic overlap between psychiatric disorders and disorders of the immune system (Michel et al. 2012). Genes related to the immune system exert various effects on synaptic pruning and plasticity, as well as the activity of microglia. Previous studies have shown excessively pruned synapses in SCZ and inappropriately pruned ones in ASD, which are believed to occasionally lead to excessive synapse formation (Liu et al. 2017). In synaptic pruning, microglia have been reported to play an important role (Frick et al. 2013) and a twin study revealed that microglia activation was related to the etiology of SCZ (Johansson et al. 2017).

It has also been shown that the other glial cell, the oligodendrocyte, can additionally contribute to the etiology of SCZ and ASD. For example, studies that examined the disrupted brain connectivity hypothesis in SCZ have provided evidence on impaired white matter tract integrity, decreased numbers of oligodendrocytes in the prefrontal cortex and hippocampus, along with reduced oligodendrocyte-related gene expression. Moreover, an oligodendrocyte deficit can cause impaired myelination with a subsequent lack of nerve impulse transmission and cognitive impairment related to SCZ. It has also been hypothesized that inflammation in the brain along with microglia overactivity is involved in oligodendrocyte dysfunction (Raabe et al. 2018, 2019).

Other studies have additionally tested this hypothesis using neuronal iPS cells. Windrem et al. (2017) transplanted glial progenitor cells generated from iPS cells into mouse brains to generate humanized glial chimeric mice. These mice exhibited both reduced white matter and myelination as compared to controls, along with reduced prepulse suppression, and abnormal behaviors such as excessive anxiety, antisocial characteristics, and sleep disturbances. This study has provided support for the presence of impaired glial maturation during the development of SCZ.

Although the 2D culture models of iPS cells are suitable for generating relatively homogeneous cell populations, this can be disadvantageous when examining the function of the neural network that connects the multiple cell types across different brain regions. Although the use of organoids in a 3D culture model can be advantageous for examining the relationships and gene expression of multiple cells, there are some limitations in studies using organoid that include: (1) cells from tissues other than the brain may sometimes be contaminated, (2) due to different embryonal origins, microglia cannot be included in almost all of the iPSC cell-derived brain models and (3) conditions arising from long-lasting processes such as aging and maturation are difficult to reproduce when using brain organoids (Quadrato et al. 2016; Raabe et al. 2018, 2019).

Future development of brain organoids will additionally allow for more detailed examinations of oligodendrocyte and microglia functions in the brain (Di Lullo and Kriegstein 2017; Koo et al. 2019). Therefore, etiological studies using brain organoids derived from MZs with different presentations of mental disorders are anticipated in the future.

Hopefully, these new types of research approaches may help to uncover useful insights into psychiatric disorders, including ASD and SCZ.

## Conclusion

As previously discussed, there have been a large number of twin studies that have examined SCZ and ASD for an extended period of time. Epidemiological twin studies on psychiatric disorders that have been used to estimate heritability have shown that there is a large genetic influence on the onset of SCZ and ASD. Although biological twin studies for psychiatric disorders have attempted to detect genetic or epigenetic differences between MZs, a consensus for these findings has yet to be definitively perfected.

We consider the advantages of twin studies to be as follows: (1) when genome-wide association studies of psychiatric disorders are performed using extra-large sample sizes, the genetic heterogeneity makes it difficult to achieve clear results that are close to the etiology of the disorder. However, if a twin study is conducted to determine genetic or epigenetic differences between the twins, then it becomes possible to obtain definitive twin-specific findings. (2) Twin studies can be undertaken with a lower cost and manpower requirement as compared to extra-large sample sizes required when performing non-twin studies. (3) Because of the uniqueness of the participants, the subjects of twin studies may be comparatively easy to follow. Therefore, twin studies can sometimes be developed into longitudinal studies.

Based on these perceived advantages, which direction should twin studies be developed? While it is first important to increase the number of samples for twin studies, this does create some limitations. Second, the definition of “discordance” needs to be discussed. For example, the discordance of severity, age of onset, the profile of symptoms, course or prognosis, response to a specific drug might potentially need to be considered. Third, new technology that makes it possible to detect differences in the MZs with regard to genetic or epigenetic states, which includes somatic mutation, DNA methylation, histone chemical modification, non-coding RNA, or neural circuits remodeling, which includes synapse formation, synaptic pruning, microglial regulation, oligodendrocyte function and myelination, will need to be developed.

For a long period of time, MZs and DZs have played an important role when attempting to study the influences of genetic and environmental factors. Recently, new concepts have been developed, which include environmental factors that are internally based such as intestinal flora, and environment-related but transgenerational factors, which include maternally inherited histone protein modification “H3K27me3”. However, researching discordant MZs remains a solid way for clarifying the etiology of psychiatric disorders. Research into MZs is expected to become more sophisticated as technological advancements make it possible to better detect the biological differences between MZs discordant for SCZ or ASD.
